# Photo-thermoelastic diffusive waves with microconcentration in quantum-modified semiconductors

**DOI:** 10.1371/journal.pone.0350100

**Published:** 2026-06-01

**Authors:** Amsawrah M. Mohammed, Eman Ghareeb Rezk, A. H. El -Sharif, Alaa A. El-Bary, Khaled Lotfy

**Affiliations:** 1 Mathematics Department, School of Basic Sciences, Libyan Academy for Graduate Studies, Ajdabia, Libya; 2 Mathematical Science Department, College of Science, Princess Nourah bint Abdulrahman University, Riyadh, Saudi Arabia; 3 Department of Mathematics, Faculty of Science, Benghazi University, Benghazi, Libya; 4 Arab Academy for Science, Technology and Maritime Transport, Alexandria, Egypt; 5 Department of Mathematics, Faculty of Science, Zagazig University, Zagazig, Egypt; Purdue University, UNITED STATES OF AMERICA

## Abstract

This study presents a general one-dimensional analysis of photo-thermoelastic diffusive wave propagation in quantum-modified semiconductor media that incorporates microconcentration effects. The model extends classical photo-thermoelastic semiconductor theory by introducing a coupled dual-transport mechanism that accounts for quantum-modified carrier diffusion and thermodiffusion associated with microconcentration fields. Quantum transport is represented through a density-gradient formulation, enabling the capture of nonlocal carrier behavior at small length scales, while the microconcentration variable describes additional mass transport induced by temperature gradients. The governing equations for displacement, temperature, carrier density, and microconcentration are formulated within a unified continuum framework and reduced to dimensionless form in a one-dimensional configuration. The resulting system is solved analytically using the Laplace transform, and the physical fields are obtained in the time domain via numerical inversion. The analysis reveals that the interaction between quantum carrier transport and thermodiffusion significantly alters the propagation characteristics, leading to modified attenuation, phase behavior, and wave penetration depth. Furthermore, microconcentration introduces additional coupling that redistributes thermal and mechanical fields within the medium. The proposed formulation provides a comprehensive tool for understanding coupled transport phenomena in semiconductor structures and is relevant to applications in optoelectronic devices, nano-scale thermal management, and laser-driven material systems.

## 1. Introduction

The interaction between thermal, mechanical, and mass transport processes plays a fundamental role in the behavior of semiconductor materials subjected to external excitation, particularly in laser-driven and micro-scale applications. In such environments, temperature gradients not only induce elastic deformation but also generate carrier redistribution and mass diffusion, leading to strongly coupled multiphysics phenomena. Classical thermoelastic theories, although successful in describing heat-deformation interactions, are inadequate for capturing the complex interplay between thermal waves, diffusive transport, and microstructural effects observed in modern semiconductor systems. Recent advances have highlighted the importance of incorporating diffusion mechanisms, especially those associated with microconcentration, to describe mass transport driven by temperature gradients and internal material structure. At the same time, the miniaturization of electronic and optoelectronic devices has brought quantum-scale effects to the forefront, where carrier transport deviates from classical diffusion due to nonlocal interactions and wave-like behavior. Consequently, a unified theoretical framework that simultaneously accounts for thermoelastic coupling, quantum-modified carrier transport, and thermodiffusion with microconcentration is essential for accurately describing wave propagation and energy transport in advanced semiconductor media.

The study of thermoelastic interactions has attracted significant attention due to its fundamental role in describing the coupling between thermal and mechanical fields in solid media. The classical theory established by Biot [1] laid the foundation for thermomechanical coupling; however, it predicts an unrealistic infinite speed of heat propagation. To address this limitation, generalized thermoelastic theories were developed, including the model of Lord and Shulman [2], which introduces a single thermal relaxation time, and the theory of Green and Lindsay [3], which incorporates two relaxation parameters. Further advancements were made by Green and Naghdi [4,5], who proposed formulations capable of describing undamped thermal wave propagation. In addition, the unified heat conduction framework introduced by Tzou [6] extended classical models to micro- and nano-scale heat transport. These developments have significantly enhanced the ability of thermoelastic theory to describe realistic thermal wave behavior.

With the increasing demand for modeling complex materials, thermoelastic theories have been extended to include additional physical effects such as porosity, microstructure, rotation, and electromagnetic interactions [7–10]. These generalized formulations have been successfully applied to porous and microstructured media, where internal material characteristics strongly influence wave propagation and energy dissipation [11,12]. Moreover, the incorporation of fractional-order derivatives and memory-dependent heat conduction models has provided improved descriptions of thermal processes with hereditary behavior, particularly in heterogeneous and advanced materials [13–16]. Such developments have broadened the applicability of thermoelasticity to a wide range of engineering and physical problems [17,18].

In parallel, diffusion theory has emerged as an essential extension of thermoelasticity, accounting for the transport of mass particles driven by temperature gradients. The coupling between thermal and diffusion fields leads to thermoelastic diffusion phenomena, which play a crucial role in materials science, geophysics, and microstructured solids [19–22]. More refined models have introduced microconcentration effects to capture microscale transport mechanisms and internal structural heterogeneity, providing a more accurate description of mass diffusion processes [23,24]. These studies demonstrated that diffusion significantly affects stress distribution, attenuation behavior, and wave propagation characteristics, particularly in transient and thermally loaded systems.

On the other hand, semiconducting materials have become a major focus of thermoelastic research due to their strong sensitivity to thermal excitation and carrier transport. Early investigations into photothermal effects revealed the interaction between thermal waves and electronic carriers under laser irradiation [25–27]. This led to the development of photo-thermoelastic semiconductor theories, which describe the coupling between elastic deformation, heat conduction, and carrier dynamics [28–32]. Recent studies have further extended these models by incorporating nonlocal effects, fractional heat conduction, plasma waves, and multi-temperature theories to better represent semiconductor behavior at micro- and nano-scales [33–37]. These works confirm that thermal, elastic, and carrier fields are strongly coupled in high-frequency and high-energy applications [38,39].

At nanoscale dimensions, classical carrier diffusion models become insufficient due to the emergence of quantum mechanical effects. To overcome this limitation, quantum corrections based on density-gradient (Bohm potential) formulations have been introduced, allowing the inclusion of nonlocal carrier transport within a continuum framework [40–42]. These models capture important phenomena such as carrier spreading, tunneling effects, and modified recombination dynamics, which significantly influence thermoelastic responses in semiconductor materials. However, most existing studies consider either quantum carrier transport or thermoelastic diffusion separately, and the combined influence of quantum effects and microconcentration-driven diffusion within a unified photo-thermoelastic framework remains largely unexplored [43–45].

Recent studies have further extended generalized thermoelasticity by incorporating fractional kernels, nonlocality, strain-gradient effects, and multiphysics coupling. Abouelregal et al. [46] developed a fractional dual-phase-lag thermoelastic model with a nonsingular Rabotnov kernel for a rotating stressed medium with a spherical cavity, demonstrating the importance of memory-dependent heat conduction in complex thermoelastic wave problems. Selvamani et al. [47] investigated the free vibration of functionally graded magneto-piezo-thermoelastic ceramic–metal nanobeams using a modified nonlocal state-space strain-gradient theory, showing that size-dependent and material-gradient effects strongly influence nanoscale structural responses. In another recent contribution, Selvamani et al. [48] analyzed fractional nonlocal couple-stress waves in magnetoelastic nanobeams using the homotopy perturbation technique, confirming the relevance of fractional damping, magnetic interaction, and nonlocal elasticity in nanostructured media. Abouelregal et al. [49] also proposed a fractional thermoelastic model for porous materials with cylindrical cavities and voids using a modified space-time-nonlocality kernel, emphasizing the role of spatial and temporal nonlocal effects in porous thermoelastic responses. Moreover, Abouelregal et al. [50] studied temperature changes in living tissue under laser heat flux using a modified fractional thermal conduction model, highlighting the importance of laser-induced thermal transport in continuum systems. These recent works confirm the growing importance of fractional, nonlocal, and multiphysics thermoelastic models; however, the combined influence of quantum-modified carrier diffusion and microconcentration-driven thermodiffusion in photo-thermoelastic semiconductor media remains insufficiently explored. This gap motivates the present study.

Recent advances in generalized thermoelasticity have emphasized the importance of finite thermal wave speeds, memory-dependent heat conduction, and nonlocal interactions in accurately describing multiphysics behavior in advanced materials. For instance, Chandel et al. [51] investigated Moore–Gibson–Thompson thermodiffusion dynamics in fractal tumor media, demonstrating the role of kernel-based memory and nonlocal effects in capturing realistic thermal responses. Similarly, Karde et al. [52] examined heat transfer in nonsimple media using a Goufo–Caputo kernel, highlighting the influence of nonlocality on thermal wave propagation. More recently, Chandel et al. [53] proposed a spatio-temporal nonlocal magneto-thermoelastic framework incorporating Gurtin–Pipkin–Moore–Gibson–Thompson heat conduction, confirming the significance of memory-dependent models in complex materials. In the context of semiconductor systems, Lute et al. [54] explored photothermoelastic behavior in fractal media using noninteger-dimensional formulations, while Lute et al. [55] further developed advanced thermoelastic models for photothermal heat generation in semiconductor plates. These studies collectively highlight the growing need for unified frameworks that incorporate nonlocality, memory effects, and multiphysics coupling. Motivated by these developments, the present work extends photo-thermoelastic modeling by integrating quantum-modified carrier transport and microconcentration-driven diffusion within a unified continuum framework.

Several recent studies have further advanced the analysis of thermoelastic media by incorporating rotation, nonlocality, memory-dependent derivatives, and multi-temperature effects. For example, Said [56] investigated the deformation of a rotating two-temperature generalized magneto-thermoelastic medium with internal heat sources, demonstrating the influence of initial stress and electromagnetic coupling on thermoelastic behavior. In a related study, Said [57] analyzed a two-dimensional nonlocal rotating thermoelastic half-space using a memory-dependent derivative, highlighting the role of nonlocality and temporal memory in wave propagation. Furthermore, Said [58] developed a fractional heat transfer model for rotating magneto-thermoelastic media within a modified couple stress framework, showing the importance of microstructural effects and fractional dynamics. Additionally, Said [59] examined the combined influence of phase lags, rotation, and temperature-dependent properties on wave propagation in magneto-microstretch thermoelastic materials. These studies demonstrate the growing importance of incorporating advanced theoretical features such as nonlocality, rotation, and memory effects. However, the combined influence of quantum-modified carrier transport and microconcentration-driven diffusion in photo-thermoelastic semiconductor media remains largely unexplored, which motivates the present investigation.

Motivated by the above developments, the present study aims to develop a comprehensive model for photo-thermoelastic diffusive wave propagation in quantum-modified semiconductors incorporating microconcentration effects. The proposed formulation integrates thermoelasticity, quantum carrier transport, and thermodiffusion into a unified framework, enabling the investigation of coupled wave behavior under decay-type thermal excitation. The problem is formulated in a one-dimensional configuration and solved analytically using the Laplace transform technique to obtain the transient response of the physical fields. This work provides new insights into multiphysics wave interactions and contributes to the advancement of semiconductor modeling in modern engineering applications.

The novelty of the present study lies in the development of a unified photo-thermoelastic framework that simultaneously incorporates quantum-modified carrier transport and thermodiffusion with microconcentration effects within a single continuum model. Unlike existing photo-thermoelastic semiconductor formulations, which primarily focus on carrier diffusion alone, the proposed model introduces an additional diffusive field representing microconcentration, thereby enabling a dual-transport description of mass transfer processes. Furthermore, quantum effects are included through a density-gradient correction, allowing nonlocal carrier behavior to influence the thermoelastic response indirectly through carrier–lattice coupling. This combined approach reveals new physical mechanisms governing wave propagation, including enhanced attenuation, modified phase velocity, and redistribution of thermal and stress fields due to the interaction between quantum transport and thermodiffusion. In addition, the use of a one-dimensional analytical formulation solved via the Laplace transform provides an efficient and transparent method for capturing transient behaviors that are often difficult to resolve using purely numerical techniques. To the best of the authors’ knowledge, the integration of quantum carrier effects, microconcentration-driven diffusion, and photo-thermoelastic coupling under decay heating has not been previously reported. The proposed model, therefore, offers a novel and comprehensive tool for analyzing multiphysics interactions in semiconductor materials and can be applied to the design and optimization of advanced optoelectronic and nano-scale devices.

The present model offers several important advancements over traditional thermoelastic formulations. Unlike classical models, which assume Fourier heat conduction and neglect carrier transport, the current framework incorporates quantum-modified carrier diffusion through a density-gradient formulation, enabling the capture of nonlocal effects that become significant at micro- and nano-scales. In addition, the inclusion of microconcentration introduces an additional diffusion mechanism that accounts for internal mass transport driven by temperature and carrier interactions, which is not considered in conventional thermoelastic or semiconductor models. Furthermore, the proposed formulation provides a fully coupled description of thermal, mechanical, and carrier fields within a single analytical framework, allowing direct investigation of their mutual interactions. The use of exponentially decaying thermal loading also offers a more realistic representation of laser-induced excitation compared to idealized thermal shocks commonly used in earlier studies. As a result, the present model is capable of describing enhanced diffusion depth, modified attenuation behavior, and stronger multiphysics coupling, thereby providing a more accurate and physically consistent representation of thermoelastic wave propagation in advanced semiconductor materials.

## 2. Governing equations of the coupled diffusive photo-thermoelastic model

### 2.1. Physical model and assumptions

The present model is developed under a set of physically reasonable assumptions to ensure analytical tractability while retaining the essential multiphysics behavior. The semiconductor medium is assumed to be homogeneous, isotropic, and linearly elastic, and the analysis is restricted to a one-dimensional configuration. Small deformations are considered, allowing the use of linear strain–displacement relations. The temperature variations are assumed to be sufficiently small so that material properties remain constant, except where explicitly modified through the coupling terms. The carrier density is treated as a small perturbation about its equilibrium value, enabling linearization of the quantum density-gradient term. The quantum correction is incorporated through the density-gradient (Bohm potential) model, which introduces nonlocal carrier transport effects without resorting to a full quantum mechanical formulation. The microconcentration field represents an additional diffusive mechanism driven by temperature and carrier interactions. The thermal loading is modeled as an exponentially decaying surface heat flux, representing laser-induced excitation. Homogeneous initial conditions are assumed, and all physical fields are required to remain bounded as the spatial coordinate tends to infinity. Finally, the solution is obtained using the Laplace transform technique, which converts the time-dependent problem into a system of ordinary differential equations in space.

In this work, a generalized theoretical model is formulated to describe the coupled behavior of elastic deformation, heat conduction, quantum-modified carrier transport, and thermodiffusion, including microconcentration effects, in a semiconductor medium. Consider a homogeneous, isotropic, linearly elastic semiconductor medium occupying a one-dimensional region 0≤x≤L. The medium is subjected to photo-thermal excitation and exhibits coupled elastic deformation, heat conduction, carrier diffusion, and microconcentration-based thermodiffusion. Let $u_{i}$ be the displacement vector, $\theta =T-T_{\text{0}}$the temperature increment ($T_{\text{0}}\;is\;$ reference temperature), $N=n-n_{\text{0}}$ the excess carrier density (n represents the carrier concentration and $n_{\text{0}}$ gives to the reference carrier concentration), and C the microconcentration/diffusive variable. The present formulation extends the quantum-modified semiconductor model by incorporating microconcentration-based diffusion effects inspired by thermoelastic diffusion theory.

### 2.2. Strain–displacement and constitutive relations

Strain-displacement relation is [1–3]:


$$\begin{array}{c} e_{ij}=\frac{\text{1}}{\text{2}}(\frac{\partial u_{i}}{\partial x_{j}}+\frac{\partial u_{j}}{\partial x_{i}}). \end{array}$$ (1)

This equation defines the infinitesimal strain tensor $e_{ij}$, while $e_{kk}$ represents the volumetric dilatation of the semiconductor medium.

In general, the constitutive relation can be written as [60]:


$$\begin{array}{c} \sigma_{ij}=\lambda e_{rr}\delta_{ij}+2\mu e_{ij}-(\gamma \theta -\delta_{n}N-\delta_{C}C)\delta_{ij}. \end{array}$$ (2)

This relation shows that the stress field is generated not only by elastic strain, but also by temperature variation, excess carrier density, and microconcentration. Here, λ and μ are Lamé constants, $\gamma =(3\lambda +2\mu )\alpha_{\theta }$ gives the thermoelastic coupling coefficient, $\delta_{n}=(3\lambda +2\mu )d_{n}$ is the carrier–elastic coupling coefficient, $\delta_{C}=(3\lambda +2\mu )\alpha_{C}$ is the diffusion–elastic coupling coefficient and $\delta_{ij}$ Kronecker delta. On the other hand, $\alpha_{\theta }$ denotes the coefficient of thermal expansion, $d_{n}$ is the difference of the deformation potential constant and $\alpha_{C}$ represents the coefficient of the linear diffusion equation.

### 2.3. Equation of motion

This equation represents the balance of linear momentum, showing how mechanical waves propagate under the influence of thermal, carrier, and diffusive fields [61]:


$$\begin{array}{c} (\lambda +\mu )\nabla (\nabla \cdot u_{i})+\mu \nabla^{\text{2}}u_{i}-\gamma \nabla \theta -\delta_{n}\nabla N-\delta_{C}\nabla C=\rho \frac{\partial^{\text{2}}u_{i}}{\partial t^{\text{2}}}. \end{array}$$ (3)

This equation governs elastic wave propagation under the influence of thermal gradients, carrier-density gradients, and microconcentration gradients, here ρ gives the medium density.

### 2.4. Heat conduction equation

This equation governs the evolution of temperature by accounting for heat diffusion, thermoelastic coupling, carrier recombination, microconcentration contribution, and external thermal excitation [62]:


$$\begin{array}{c} \rho C_{E}\frac{\partial \theta }{\partial t}=K\nabla^{\text{2}}\theta +\gamma T_{o}\frac{\partial }{\partial t}(\nabla .u_{i})+\frac{E_{g}}{\tau }N+cT_{\text{0}}C. \end{array}$$ (4)

This equation describes heat transfer in the semiconductor. Here $C_{E}$ gives the specific heat at constant strain, K denotes the thermal conductivity, $\nabla^{\text{2}}$ represents the Laplacian operator; $E_{g}$ is the energy band gap, c denotes the thermoelastic diffusion effect coefficient and τ gives the carrier recombination time.

### 2.5. Quantum-modified carrier diffusion equation

The quantum-modified carrier diffusion equation represents the evolution of excess charge carriers in the semiconductor under the combined influence of classical diffusion, recombination processes, thermal coupling, and microconcentration interaction. This equation describes the transport of excess carriers, incorporating classical diffusion, recombination, thermal coupling, microconcentration interaction, and quantum nonlocal effects [63]:


$$\begin{array}{c} \frac{\partial N}{\partial t}=D_{E}\nabla^{\text{2}}N-\text{\textbackslash{}hspace\{0.17em}\}\frac{N}{\tau }+\kappa \theta +\nabla .J_{q}, \end{array}$$ (5)

here $D_{E}$ denotes the carrier diffusion coefficient, κ is the thermal-carrier coupling coefficient, and $J_{q}$ denotes the quantum flux arising from the density gradient (Bohm potential) formulation, capturing nonlocal carrier transport effects that become significant at micro- and nano-scales.

### 2.6. Quantum correction via density-gradient model

To account for quantum mechanical effects in carrier transport, a density-gradient (Bohm potential) correction is incorporated into the carrier diffusion equation. This approach provides a continuum-level description of quantum phenomena such as carrier confinement, nonlocal transport, and wave-like spreading, which become significant when the characteristic length scale of the semiconductor is comparable to the carrier de Broglie wavelength. The quantum contribution is introduced through the quantum flux vector $J_{q}$, defined as [64]:


$$\begin{array}{c} J_{q}=-\frac{{\text{ℏ}}^{\text{2}}}{2mn_{\text{0}}}\nabla \left(\frac{\nabla^{\text{2}}\sqrt{N}}{\sqrt{N}}\right), \end{array}$$ (6)

where ℏ denotes the reduced Planck constant, and m gives the effective carrier mass [48]. This expression originates from the Bohm potential in quantum hydrodynamics and represents the effect of quantum pressure acting on the carrier distribution. For small perturbations around the equilibrium state, the carrier density is expressed as $N=n_{\text{0}}+n$, $n\ll n_{\text{0}}$. By linearizing the quantum flux under this assumption, the divergence of $J_{q}$ can be approximated as:


$$\begin{array}{c} \nabla .J_{q}\approx Q_{N}\nabla^{\text{4}}N, \end{array}$$ (7)

where $Q_{N}=\frac{{\text{ℏ}}^{\text{2}}}{4mn_{\text{0}}}$ is the quantum coefficient. Substituting this expression into the carrier diffusion equation yields a fourth-order spatial derivative term, which captures the nonlocal nature of quantum transport:


$$\begin{array}{c} \frac{\partial N}{\partial t}=D_{E}\nabla^{\text{2}}N-\text{\textbackslash{}hspace\{0.17em}\}\frac{N}{\tau }+\kappa \theta +\frac{{\text{ℏ}}^{\text{2}}}{4mn_{\text{0}}}\nabla^{\text{4}}N. \end{array}$$ (8)

Physically, this correction leads to smoother carrier concentration profiles, reduced localization near interfaces, and increased penetration depth compared to classical diffusion models. Unlike fully quantum mechanical approaches, the density-gradient model maintains analytical tractability while retaining the essential features of quantum carrier dynamics, making it particularly suitable for studying wave propagation in semiconductor media.

### 2.7. Microconcentration diffusion equation

The evolution of the microconcentration field Cis governed by a generalized thermodiffusion equation that accounts for the combined effects of diffusion, thermal gradients, carrier interaction, and deformation-induced transport. In its general form, the equation can be written as [50]:


$$\begin{array}{c} D_{c}\gamma_{C}e_{nn,ii}+D_{c}c\theta_{,ii}\left(r,t\right)+\frac{\partial C}{\partial t}=D_{c}bC_{,ii}, \end{array}$$ (9)

where $D_{c}$ gives the microconcentration diffusion coefficient and b is a measure of the diffusive influence. This equation describes the transport of mass at the microscale, driven not only by concentration gradients but also by temperature variations and carrier redistribution. In thermoelastic diffusion theory with microconcentration effects, the chemical potential represents the driving force for mass transport and is influenced by concentration, temperature, and deformation. A generalized linear form of the chemical potential can be expressed as: $P=-\gamma_{C}e_{nn}+bC-c\theta$, where P gives the chemical potential of the material per unit mass and $e_{nn}$is the volumetric strain (dilatation). This equation defines the chemical potential as a function of microconcentration, temperature variation, and volumetric strain.

**Fig 1:**
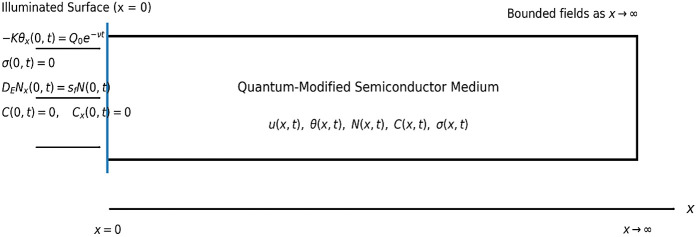
The one-dimensional schematic of the problem.


The displacement field is assumed to act along the x-direction only, so that u=u(x,t). The temperature increment is denoted by θ(x,t), the excess photo-generated carrier density by N(x,t), and the microconcentration/diffusive field by C(x,t). Fig 1. Schematic representation of the one-dimensional quantum photo-thermoelastic diffusive model. The semiconductor medium occupies the semi-infinite domain x≥0 and is subjected to a decay-type thermal excitation at the illuminated surface. Mechanical, thermal, carrier, and microconcentration boundary conditions are applied at x=0, while all fields remain bounded as x→∞. For one-dimensional deformation, the main equations are reduced to:

$$\begin{array}{c} \sigma =\sigma_{xx}=\text{(2}\mu +\lambda \text{)}\frac{\partial u}{\partial x}-(3\lambda +2\mu )(\alpha_{\theta }\text{\textbackslash{}hspace\{0.17em}\}\theta \text{\textbackslash{}hspace\{0.17em}\}+d_{n}N+\alpha_{C}C),\text{\textbackslash{}hspace\{0.17em}\}e=\frac{\partial u}{\partial x}, \end{array}$$ (10)

$$\begin{array}{c} (\lambda +2\mu )\frac{\partial^{\text{2}}u}{\partial x^{\text{2}}}-\gamma \frac{\partial \theta }{\partial x}-\delta_{n}\frac{\partial N}{\partial x}-\delta_{C}\frac{\partial C}{\partial x}=\rho \frac{\partial^{\text{2}}u}{\partial t^{\text{2}}}, \end{array}$$ (11)

$$\begin{array}{c} \rho C_{E}\frac{\partial \theta }{\partial t}=K\frac{\partial^{\text{2}}\theta }{\partial x^{\text{2}}}+\gamma T_{o}\frac{\partial e}{\partial t}+\frac{E_{g}}{\tau }N+cT_{\text{0}}C, \end{array}$$ (12)

$$\begin{array}{c} \frac{\partial N}{\partial t}=D_{E}\frac{\partial^{\text{2}}N}{\partial x^{\text{2}}}-\text{\textbackslash{}hspace\{0.17em}\}\frac{N}{\tau }+\kappa \theta +Q_{N}\frac{\partial^{\text{4}}N}{\partial x^{\text{4}}}, \end{array}$$ (13)

$$\begin{array}{c} D_{c}\gamma_{C}\frac{\partial^{\text{2}}e}{\partial x^{\text{2}}}+D_{c}c\frac{\partial^{\text{2}}\theta }{\partial x^{\text{2}}}+\frac{\partial C}{\partial t}=D_{c}b\frac{\partial^{\text{2}}C}{\partial x^{\text{2}}}, \end{array}$$ (14)

It is important to clarify the mathematical structure of the governing system in terms of spatial and temporal orders. The elastic displacement equation (Eq. 3) is second-order in both space and time, reflecting the wave-like nature of mechanical propagation. The heat conduction equation (Eq. 4) is second-order in space and first-order in time, consistent with generalized heat diffusion models. The classical carrier diffusion equation is also second-order in space and first-order in time. However, the inclusion of the quantum correction through the density-gradient formulation (Eq. 8) introduces a fourth-order spatial derivative term, transforming the carrier transport equation into a higher-order diffusion model.

This fourth-order spatial term represents nonlocal carrier interactions and quantum pressure effects, which become significant at micro- and nano-scales. From a mathematical standpoint, the presence of this higher-order derivative increases the overall order of the coupled system. Consequently, after eliminating the dependent variables, the resulting characteristic equation becomes of higher degree (tenth order in the present formulation), which directly reflects the combined influence of thermoelastic coupling, diffusion processes, and quantum nonlocality. This elevated order is responsible for the richer wave behavior and additional attenuation modes observed in the numerical results.

### 2.8. Special Cases

The limiting forms of the proposed formulation are summarized in Table 1, where several classical and generalized models are recovered under specific simplifying assumptions.

**Table 1:** Special Cases of the Present Model.

Case No.	Assumption	Reduced Model	Physical Description
1 [1 –3]	N=C=0	Classical thermoelasticity	Describes coupling only between displacement and temperature fields without diffusion or semiconductor effects.
2 [23, 24]	C=0	Quantum photo-thermoelastic semiconductor model	Represents interaction between elastic, thermal, and carrier fields with quantum transport effects included.
3 [8, 9]	N=0	Thermoelastic diffusion with microconcentration	Describes coupled thermal, mechanical, and diffusive behavior due to microconcentration transport.
4 [48, 49]	$Q_{N}=0$	Classical carrier diffusion model	Eliminates quantum effects, reducing carrier transport to classical diffusion–recombination behavior.
5 [24]	u=θ=0	Pure diffusion model	Represents microconcentration evolution independent of thermoelastic and carrier interactions.
6 [1 –6]	N=C=u=0	Fourier heat conduction model	Describes uncoupled heat diffusion governed solely by thermal conduction.

In the limiting case where the quantum parameter tends to zero, the fourth-order density-gradient contribution disappears and the carrier diffusion equation reduces to the classical second-order diffusion model. Consequently, the natural quantum-flux boundary condition becomes redundant, while the standard surface recombination condition and boundedness requirement remain sufficient to close the classical carrier boundary-value problem. Hence, the proposed quantum boundary formulation reduces smoothly to the classical carrier diffusion case.

To facilitate the analytical treatment and reduce the number of governing parameters, the basic field equations are transformed into a dimensionless form using the characteristic scales defined as follows:


$$\begin{array}{c} \left(x^{\prime },u^{\prime }\right)=\frac{\left(x,u\right)}{C_{T}t^{*}},\text{\textbackslash{}hspace\{0.17em}\;\}t^{\prime }=\frac{t}{t^{*}},\text{\textbackslash{}hspace\{0.17em}\}\theta^{\prime }=\frac{\gamma \theta }{2\mu +\lambda },\text{\textbackslash{}hspace\{0.17em}\}C^{\prime }=\frac{\delta_{C}C}{2\mu +\lambda },\text{\textbackslash{}hspace\{0.17em}\}\sigma^{\prime }=\frac{\sigma }{\mu },\text{\textbackslash{}hspace\{0.17em}\}P^{\prime }=\frac{P}{\delta_{\text{C}}},\text{\textbackslash{}hspace\{0.17em}\}N^{\prime }=\frac{\delta_{n}N}{2\mu +\lambda }. \end{array}$$ (15)

As a result of equation (15), the governing equations of motion, heat conduction, quantum carrier diffusion, and microconcentration transport are rewritten in a compact nondimensional form (the primes may be omitted for simplicity):


$$\begin{array}{c} \frac{\partial^{\text{2}}u}{\partial x^{\text{2}}}-\frac{\partial \theta }{\partial x}-\frac{\partial N}{\partial x}-\frac{\partial C}{\partial x}=\frac{\partial^{\text{2}}u}{\partial t^{\text{2}}}, \end{array}$$ (16)

$$\begin{array}{c} \frac{\partial \theta }{\partial t}=\varepsilon_{\text{1}}\frac{\partial^{\text{2}}\theta }{\partial x^{\text{2}}}+\varepsilon_{\text{2}}\frac{\partial e}{\partial t}+\varepsilon_{\text{3}}N+\varepsilon_{\text{4}}C, \end{array}$$ (17)

$$\begin{array}{c} \frac{\partial N}{\partial t}=q_{\text{1}}\frac{\partial^{\text{2}}N}{\partial x^{\text{2}}}-q_{\text{2}}\text{\textbackslash{}hspace\{0.17em}\}N+q_{\text{3}}\theta +q_{\text{4}}\frac{\partial^{\text{4}}N}{\partial x^{\text{4}}}, \end{array}$$ (18)

$$\begin{array}{c} \eta_{\text{1}}\frac{\partial^{\text{2}}e}{\partial x^{\text{2}}}+\eta_{\text{2}}\frac{\partial^{\text{2}}\theta }{\partial x^{\text{2}}}+\frac{\partial C}{\partial t}=\eta_{\text{3}}\frac{\partial^{\text{2}}C}{\partial x^{\text{2}}}, \end{array}$$ (19)

where

$\text{\textbackslash{}hspace\{0.17em}\}\varepsilon_{\text{1}}=\frac{K}{\rho \overset{\text{2}}{\underset{T}{C}}C_{E}t^{*}},$ $\text{\textbackslash{}hspace\{0.17em}\}\varepsilon_{\text{2}}=\frac{\gamma^{\text{2}}T_{\text{0}}}{\rho C_{E}(\lambda +2\mu )}$, $\text{\textbackslash{}hspace\{0.17em}\}\varepsilon_{\text{3}}=\frac{\gamma E_{g}t^{*}}{\delta_{n}\rho \tau C_{E}}$, $\varepsilon_{\text{4}}=\frac{\gamma cT_{\text{0}}t^{*}}{\delta_{C}\rho C_{E}}$, $\text{\textbackslash{}hspace\{0.17em}\}q_{\text{1}}=\frac{D_{E}}{t^{*}\overset{\text{2}}{\underset{T}{C}}}$, $\text{\textbackslash{}hspace\{0.17em}\}q_{\text{2}}=\frac{t^{*}}{\tau }$, $q_{\text{3}}=\frac{\delta_{n}\kappa t^{*}}{\gamma }$, $q_{\text{4}}=\frac{Q_{N}}{(t^{*}{)}^{\text{3}}\overset{\text{4}}{\underset{T}{C}}}$$\text{\textbackslash{}hspace\{0.17em}\}t^{*}=\frac{k}{\rho C_{E}\overset{\text{2}}{\underset{T}{C}}}$, $\text{\textbackslash{}hspace\{0.17em}\}\eta_{\text{1}}=\frac{\gamma_{C}D_{C}\delta_{C}}{t^{*}\overset{\text{2}}{\underset{T}{C}}(\lambda +2\mu )}$, $\text{\textbackslash{}hspace\{0.17em}\}\eta_{\text{2}}=\frac{cD_{C}\delta_{C}}{t^{*}\overset{\text{2}}{\underset{T}{C}}\gamma }$, $\text{\textbackslash{}hspace\{0.17em}\}\eta_{\text{3}}=\frac{D_{C}b}{t^{*}\overset{\text{2}}{\underset{T}{C}}}$ $\overset{\text{2}}{\underset{T}{C}}=\frac{2\mu +\lambda }{\rho }$.

The dimensionless stress equation is:


$$\begin{array}{c} \sigma =\frac{\text{(2}\mu +\lambda \text{)}}{\mu }\left(\frac{\partial u}{\partial x}-(\text{\textbackslash{}hspace\{0.17em}\}\theta \text{\textbackslash{}hspace\{0.17em}\}+N+C)\right). \end{array}$$ (20)

The quantum-modified carrier equation involves a fourth-order spatial derivative, and therefore, the carrier density must possess sufficient smoothness in space. In the classical formulation, the carrier density is assumed to be sufficiently differentiable up to fourth order, while the displacement and temperature fields require only second-order spatial smoothness, consistent with their governing equations. To ensure mathematical closure of the fourth-order carrier equation, the classical surface recombination condition is supplemented by an additional natural boundary condition associated with quantum correction. This additional condition represents the absence of externally imposed higher-order carrier flux at the surface. Together with the requirement that all physical fields remain bounded far from the boundary, these conditions provide the necessary constraints to match the order of the governing equations, ensuring that the boundary-value problem is neither underdetermined nor ill-posed. Furthermore, when the quantum parameter tends to zero, the fourth-order contribution disappears, and the carrier equation reduces smoothly to the classical second-order diffusion model. In this limiting case, the additional quantum boundary condition becomes unnecessary, and the standard surface recombination condition, together with the boundedness requirement, is sufficient. This demonstrates the consistency and mathematical well-posedness of the proposed formulation.

## 3. Laplace transform solution

To obtain the transient response of the coupled photo-thermoelastic diffusive system, the Laplace transform technique is applied with respect to time. This method is suitable for the present one-dimensional problem because it converts the time-dependent governing equations into a system of ordinary differential equations in the spatial coordinate x. Consequently, the coupled effects of elastic deformation, temperature, carrier density, and microconcentration can be treated analytically in the transformed domain.

The Laplace transform of any field variable f(x,t) is defined as:


$$\begin{array}{c} \overset{―}{f}\left(s,t\right)=L\left(f\left(x,t\right)\right)=\underset{\text{0}}{\int^{\infty }}f\left(x,t\right)exp(-st)dt,\text{\textbackslash{}hspace\{0.17em}\;\;\;\;\;\;\;\;\;\;s>0\}, \end{array}$$ (21)

where s is the Laplace transform parameter and the overbar denotes the transformed quantity. Assuming homogeneous initial conditions,


$$\begin{array}{c} {e\left(x,\text{\textbackslash{}hspace\{0.17em}\}t\right)|}_{t=0}=\text{\textbackslash{}hspace\{0.17em}\;\;\}{\frac{\partial e\left(x,\text{\textbackslash{}hspace\{0.17em}\}t\right)}{\partial t}|}_{t=0}=0,\text{\textbackslash{}hspace\{0.17em}\;\}{\theta \left(x,t\right)|}_{t=0}={\frac{\partial \theta \left(x,\text{\textbackslash{}hspace\{0.17em}\}t\right)}{\partial t}|}_{t=0}=0,\text{\textbackslash{}hspace\{0.17em}\;\}{C\left(x,t\right)|}_{t=0}={\frac{\partial C\left(x,\text{\textbackslash{}hspace\{0.17em}\}t\right)}{\partial t}|}_{t=0}=0,\text{\textbackslash{}hspace\{0.17em}\}{N\left(x,t\right)|}_{t=0}={\frac{\partial N\left(x,\text{\textbackslash{}hspace\{0.17em}\}t\right)}{\partial t}|}_{t=0}=0. \end{array}$$ (22)

The dimensionless governing equations become, in the Laplace domain:


$$\begin{array}{c} (D^{\text{2}}-s^{\text{2}})e-D^{\text{2}}(\overset{―}{\theta }+\overset{―}{N}+\overset{―}{C})=0, \end{array}$$ (23)

$$\begin{array}{c} (\varepsilon_{\text{1}}D^{\text{2}}-s)\overset{―}{\theta }+\varepsilon_{\text{2}}s\overset{―}{e}+\varepsilon_{\text{3}}\overset{―}{N}+\varepsilon_{\text{4}}\overset{―}{C}=0, \end{array}$$ (24)

$$\begin{array}{c} (q_{\text{4}}D^{\text{4}}+q_{\text{1}}D^{\text{2}}-\beta_{\text{1}})\overset{―}{N}+q_{\text{3}}\overset{―}{\theta }=0, \end{array}$$ (25)

$$\begin{array}{c} (\eta_{\text{3}}D^{\text{2}}-s)\overset{―}{C}-\eta_{\text{1}}D^{\text{2}}\overset{―}{e}-\eta_{\text{2}}D^{\text{2}}\overset{―}{\theta }=\text{0}\text{,} \end{array}$$ (26)

$$\begin{array}{c} \overset{―}{\sigma }=\beta_{\text{2}}\left(\overset{―}{e}-(\text{\textbackslash{}hspace\{0.17em}\}\overset{―}{\theta }\text{\textbackslash{}hspace\{0.17em}\}+\overset{―}{N}+\overset{―}{C})\right), \end{array}$$ (27)

$$\begin{array}{c} \overset{―}{P}=-\gamma_{C}\overset{―}{e}+b\overset{―}{C}-c\overset{―}{\theta }\text{,} \end{array}$$ (28)

where $D=\frac{d}{dx}$, $\beta_{\text{1}}=q_{\text{2}}\text{\textbackslash{}hspace\{0.17em}\}+s,\beta_{\text{2}}=\frac{\text{(2}\mu +\lambda \text{)}}{\mu }$.

Thus, the elimination of $\bar{e},\bar{N},\bar{C}$ gives the following differential equation for $\bar{\theta }$::


$$\begin{array}{c} \left(D^{10}-E_{\text{1}}D^{\text{8}}+E_{\text{2}}D^{\text{6}}-E_{\text{3}}D^{\text{4}}+E_{\text{4}}D^{\text{2}}-E_{\text{5}}\right)\theta^{*}\left(x\right)=0, \end{array}$$ (29)

here,

$E_{\text{1}}={\left(E_{\text{6}}\right)}^{-1}\left(\varepsilon_{\text{1}}q_{\text{1}}(\eta_{\text{1}}-\eta_{\text{3}})+\varepsilon_{\text{1}}q_{\text{4}}(\eta_{\text{3}}s^{\text{2}}+s)-\varepsilon_{\text{2}}\;q_{\text{4}}\;s(\eta_{\text{2}}\;+\eta_{\text{3}})-\varepsilon_{\text{4}}\;q_{\text{4}}\;(\eta_{\text{1}}+\;\eta_{\text{2}}\;)-q_{\text{4}}\;s(\eta_{\text{1}}\;-\eta_{\text{3}})\right)$$E_{\text{2}}={\left(E_{\text{6}}\right)}^{-1}\left(\begin{array}{c} @l@\beta_{\text{1}}\varepsilon_{\text{2}}s(\eta_{\text{2}}-\eta_{\text{3}})-\beta_{\text{1}}\varepsilon_{\text{1}}(\eta_{\text{3}}s^{\text{2}}+s)+\beta_{\text{1}}\varepsilon_{\text{4}}(\eta_{\text{1}}+\eta_{\text{2}})+\beta_{\text{1}}s(\eta_{\text{1}}-\eta_{\text{3}})+(\varepsilon_{\text{1}}+\eta_{\text{3}})q_{\text{1}}s^{\text{3}}+\\ (\varepsilon_{\text{2}}-\varepsilon_{\text{4}}\eta_{\text{2}}+1)q_{\text{1}}s^{\text{2}}-\varepsilon_{\text{3}}q_{\text{3}}(\eta_{\text{1}}-\eta_{\text{3}})+q_{\text{3}}(\varepsilon_{\text{4}}\eta_{\text{1}}+\varepsilon_{\text{2}}\eta_{\text{3}}s)-q_{\text{4}}s^{\text{4}}, \end{array}\right)$ $E_{\text{3}}={\left(E_{\text{6}}\right)}^{-1}\left(\begin{array}{c} @l@\beta_{\text{1}}\;\left[\varepsilon_{\text{1}}\;\left(\eta_{\text{3}}\;s^{\text{2}}+s)-\varepsilon_{\text{2}}\;s(\eta^{\text{2}}\;+\eta^{\text{3}}\;)-\varepsilon_{\text{4}}\;(\eta_{\text{1}}\;+\eta_{\text{2}}\;)+s(\eta_{\text{3}}\;-\eta_{\text{1}}\;\right)\right]+\\ \varepsilon_{\text{1}}\;q_{\text{1}}\;s^{\text{3}}-\varepsilon_{\text{2}}\;s(\eta_{\text{3}}\;q_{\text{3}}\;+q_{\text{1}}\;s)-\varepsilon_{\text{3}}\;q_{\text{3}}\;(\eta_{1\;}-\eta_{\text{3}}\;)-\varepsilon_{\text{4}}\;(\eta_{\text{1}}\;q_{\text{3}}\;+\eta_{\text{2}}\;q_{\text{1}}\;s^{\text{2}})+\eta_{\text{3}} \end{array}\right)$$E_{\text{4}}={\left(E_{\text{6}}\right)}^{-1}\left(\beta_{\text{1}}\left[-\varepsilon_{\text{1}}s^{\text{3}}+\varepsilon_{\text{2}}s^{\text{2}}+\varepsilon_{\text{4}}\eta_{\text{2}}s^{\text{2}}-\eta_{\text{3}}s^{\text{3}}-s^{\text{2}}\right]+\varepsilon_{\text{2}}q_{\text{3}}s^{\text{2}}+\varepsilon_{\text{3}}q_{\text{3}}(\eta_{\text{3}}s^{\text{2}}+s)-q_{\text{1}}s^{\text{4}}\right),$ $E_{\text{5}}\;={\left(E_{\text{6}}\right)}^{-1}s^{\text{3}}(\beta_{\text{1}}\;s-\varepsilon_{\text{3}}\;q_{\text{3}}\;)$, $E_{\text{6}}=q_{\text{4}}\varepsilon_{\text{3}}(\eta_{\text{3}}-\eta_{\text{1}})$.

Factorizing equation (29) gives:


$$\begin{array}{c} \left(D^{\text{2}}-\overset{\text{2}}{\underset{\text{1}}{k}}\right)\left(D^{\text{2}}-\overset{\text{2}}{\underset{\text{2}}{k}}\right)\text{\textbackslash{}hspace\{0.17em}\}\left(D^{\text{2}}-\overset{\text{2}}{\underset{\text{3}}{k}}\right)\left(D^{\text{2}}-\overset{\text{2}}{\underset{\text{4}}{k}}\right)\left(D^{\text{2}}-\overset{\text{2}}{\underset{\text{5}}{k}}\right)\theta^{*}\left(x\right)=0, \end{array}$$ (30)

Thus, the characteristic equation becomes:


$$\begin{array}{c} m^{10}-E_{\text{1}}m^{\text{8}}+E_{\text{2}}m^{\text{6}}-E_{\text{3}}m^{\text{4}}+E_{\text{4}}m^{\text{2}}-E_{\text{5}}=0, \end{array}$$ (31)

where $\overset{\text{2}}{\underset{n}{k}}\text{\textbackslash{}hspace\{0.17em}\}(n=1\text{\textbackslash{}hspace\{0.17em}\},2,3,4,5)$ is the characteristic root. The roots with positive real parts are selected to ensure bounded solutions inside the semiconductor medium. The admissible spatial modes are selected from the roots of the fully coupled characteristic equation obtained after applying the Laplace transform with respect to time. For the semi-infinite domain, only roots with positive real parts are retained, since these modes decay as the spatial coordinate increases and therefore satisfy the boundedness requirement. Modes with negative real parts are excluded because they lead to unbounded growth away from the illuminated surface. This root-selection criterion is also physically consistent with attenuation, energy dissipation, and the absence of nonphysical energy amplification in the coupled thermoelastic–carrier system.

Accordingly, the transformed physical temperature field can be written as a linear combination of the admissible roots $\overset{\text{2}}{\underset{n}{k}}\text{\textbackslash{}hspace\{0.17em}\}(n=1\text{\textbackslash{}hspace\{0.17em}\},2,3,4,5)$:


$$\begin{array}{c} \overset{―}{\theta }\left(x,s\right)=\sum_{n=1}^{\text{5}}G_{n}\text{\textbackslash{}hspace\{0.17em}\}exp(-k_{n}x), \end{array}$$ (32)

where $G_{n}$ are arbitrary constants determined from the boundary conditions. Therefore, the transformed physical fields can be written as:


$$\begin{array}{c} \overset{―}{N}\left(x,s\right)=\sum_{n=1}^{\text{5}}H_{1n}G_{n}\text{\textbackslash{}hspace\{0.17em}\}exp(-k_{n}x), \end{array}$$ (33)

$$\begin{array}{c} \overset{―}{e}\left(x,s\right)=\sum_{n=1}^{\text{5}}H_{2n}G_{n}exp(-k_{n}x), \end{array}$$ (34)

$$\begin{array}{c} \overset{―}{C}\left(x,s\right)=\sum_{n=1}^{\text{5}}H_{3n}G_{n}exp(-k_{n}x), \end{array}$$ (35)

Where


$$\begin{array}{c} H_{1n}=\frac{q_{\text{3}}}{q_{\text{3}}\overset{\text{4}}{\underset{n}{k}}-q_{\text{1}}\overset{\text{2}}{\underset{n}{k}}-\beta_{\text{1}}},\text{\textbackslash{}hspace\{0.17em}\;\;\;\;\;\;\;\;\;\;\;\;\;\;\;\;\;\;\;\;\;\;\}n=1,\text{\textbackslash{}hspace\{0.17em}\}2,3,4,5. \end{array}$$ (36)

$$\begin{array}{c} H_{2n}=\frac{\overset{\text{2}}{\underset{n}{k}}(1+H_{1n}+H_{3n})}{\overset{\text{2}}{\underset{n}{k}}-s^{\text{2}}},\text{\textbackslash{}hspace\{0.17em}\;\;\;\;\;\;\;\;\;\;\;\;\;\;\;\;\;\;\;\;\;\}n=1,\text{\textbackslash{}hspace\{0.17em}\}2,3,4,5. \end{array}$$ (37)

$$\begin{array}{c} H_{3n}=\frac{\overset{\text{2}}{\underset{n}{k}}(\eta_{\text{1}}H_{2n}+\eta_{\text{2}})}{\eta_{\text{3}}\overset{\text{2}}{\underset{n}{k}}-s},\text{\textbackslash{}hspace\{0.17em}\;\;\;\;\;\;\;\;\;\;\;\;\;\;\;\;\;\;\;\;\;\;\;\;\;\}n=1,\text{\textbackslash{}hspace\{0.17em}\}2,3,4,5. \end{array}$$ (38)

Since $\overset{―}{e}=D\overset{―}{u}$, the displacement is obtained as:


$$\begin{array}{c} \overset{―}{u}\left(x,s\right)=-\sum_{n=1}^{\text{5}}\frac{H_{2n}}{k_{n}}G_{n}e^{-k_{n}x}. \end{array}$$ (39)

Finally, the stress field becomes


$$\begin{array}{c} \overset{―}{\sigma }=\beta_{\text{2}}\sum_{n=1}^{\text{5}}\left(H_{2n}-1-H_{1n}\text{\textbackslash{}hspace\{0.17em}\}-H_{3n}\right)M_{n}exp\left(-k_{n}x\right). \end{array}$$ (40)

Finally, the physical fields in the time domain are recovered by applying a suitable numerical inverse Laplace transform, such as the Riemann-sum approximation.

## 4. Inverse laplace transform

After obtaining the transformed solutions $\bar{u}(x,s)$, $\bar{\theta }(x,s)$, $\bar{N}(x,s)$, $\bar{C}(x,s)$, and $\bar{\sigma }(x,s)$, the corresponding time-domain physical fields are recovered by applying the inverse Laplace transform. For a general transformed function $\bar{f}(x,s)$, the inverse Laplace transform is defined as:


$$\begin{array}{c} f\left(x,t\right)=L^{-1}\left\{\overset{―}{f}\left(x,\text{\textbackslash{}hspace\{0.17em}\}s\right)\right\}=\frac{\text{1}}{2\pi i}\int_{\Gamma -i\infty }^{\Gamma +i\infty }exp\left(st\right)\overset{―}{f}\left(x,\text{\textbackslash{}hspace\{0.17em}\}s\right)ds, \end{array}$$ (41)

where Γ is a real constant chosen so that all singularities of $\bar{f}(x,s)$ lie to the left of the vertical line s=Γ in the complex plane.

Since the transformed solutions contain complicated characteristic roots $k_{n}(s)$, direct analytical inversion is generally difficult. Therefore, a numerical inverse Laplace transform is employed. In the present work, the Riemann-sum approximation is adopted because of its efficiency and wide use in transient thermoelastic and diffusion problems. According to this method, the inverse Laplace transform of $\bar{f}(x,s)$ can be approximated as:


$$\begin{array}{c} f\left(x,\text{\textbackslash{}hspace\{0.17em}\}t\right)=\frac{e^{\Gamma t}}{t}\left[\frac{\text{1}}{\text{2}}\overset{―}{f}(x,\text{\textbackslash{}hspace\{0.17em}\}\Gamma /t)+Re\sum_{n=1}^{M}{\left(-1\right)}^{n}\overset{―}{f}\left(x,\frac{\Gamma +in\pi }{t}\right)\right], \end{array}$$ (42)

where $i=\sqrt{-\text{1}}$, Mis the number of terms used in the summation, and Γ is a convergence parameter controlling the accuracy and stability of the inversion.

The numerical implementation is carried out by evaluating the transformed functions at complex values of the Laplace parameter s, solving the corresponding characteristic equation for $m_{j}(s)$, determining the unknown constants from the boundary conditions, and then applying the above inversion formula. This procedure enables the reconstruction of the transient distributions of displacement, temperature, carrier density, microconcentration, and stress in the semiconductor medium.

## 5. Surface boundary conditions

To complete the analytical solution of the transformed governing equations, appropriate boundary conditions must be specified to determine the unknown constants appearing in the general solution. In the present one-dimensional formulation, five constants arise from the characteristic equation, and therefore five independent boundary conditions are required. These conditions are imposed at the illuminated surface of the semiconductor medium and are chosen to reflect realistic physical behavior under photo-thermal excitation. Specifically, the boundary conditions account for mechanical equilibrium at the surface, the applied heat flux due to laser or thermal loading, carrier recombination effects, and constraints on the microconcentration field. Together, these conditions ensure a well-posed boundary value problem and allow the complete determination of the displacement, temperature, carrier density, and microconcentration fields in the transformed domain. For a one-dimensional semiconductor medium occupying x≥0, the following physically meaningful boundary conditions may be used at the illuminated surface x=0:

### 5.1. Mechanical boundary conditions

This condition assumes that the surface is mechanically free, meaning that no external traction is applied at the boundary:


$$\begin{array}{c} \sigma \left(0,\text{\textbackslash{}hspace\{0.17em}\}t\right)=\text{\textbackslash{}hspace\{0.17em}\;\}0, \end{array}$$ (44)

which represents a mechanically free surface.

### 5.2. Thermal boundary conditions

This condition represents the application of a prescribed heat flux at the surface due to photo-thermal or laser excitation:


$$\begin{array}{c} K\frac{\partial \theta \left(0,t\right)}{\partial x}=Q_{\text{0}}\left(t\right), \end{array}$$ (45)

where $Q_{\text{0}}=T_{\text{0}}e^{-\upsilon t}$ represents the heat flux intensity at the surface and υ is the decay parameter of heat source.

### 5.3. Carrier boundary conditions

Since the quantum-modified carrier diffusion equation contains a fourth-order spatial derivative due to the density-gradient correction, the classical surface recombination condition alone is not sufficient to close the carrier boundary-value problem. Therefore, an additional natural quantum boundary condition is imposed at the illuminated surface. In the present model, this condition is chosen as the vanishing higher-order quantum flux, expressed by setting the third-order spatial derivative of the carrier density to zero at the surface. Physically, this means that no external quantum-pressure flux or higher-order carrier flux is prescribed at the boundary. Accordingly, the carrier boundary conditions consist of the classical surface recombination condition and the natural quantum-flux condition. Together with the boundedness condition as the spatial coordinate tends to infinity, these conditions provide the required closure for the fourth-order carrier equation and ensure that the boundary-value problem is not underdetermined. This condition describes surface recombination of charge carriers, where the carrier flux at the boundary is proportional to the carrier density:


$$\begin{array}{c} \frac{dN\left(0,t\right)}{dx}=\frac{s_{f}}{D_{E}}N\left(0,t\right), \end{array}$$ (46)

which represents surface recombination of photo-generated carriers and $s_{f}$ gives the surface recombination velocity.

Since the quantum-modified carrier diffusion equation contains the fourth-order spatial operator ($\nabla^{\text{4}}N$), the first-order surface recombination condition alone is not sufficient to close the carrier boundary-value problem. Therefore, an additional natural boundary condition associated with the quantum correction is imposed at the illuminated surface. In the absence of an externally prescribed quantum-pressure flux, the higher-order carrier flux is assumed to vanish, namely


$$\begin{array}{c} \frac{d^{\text{3}}N}{dx^{\text{3}}}\left(0,t\right)=0. \end{array}$$ (47)

The first condition represents classical surface recombination, whereas the second condition states that no external higher-order quantum carrier flux is prescribed at the surface. This condition complements the surface recombination condition and provides the additional boundary information required by the fourth-order carrier diffusion equation. The boundary conditions are mutually compatible because the higher-order requirement applies only to the quantum carrier field, and this field is now supplied with both the classical recombination condition and an additional natural quantum-flux condition. Therefore, the classical surface recombination condition governs the first-order carrier flux, while the additional quantum boundary condition accounts for higher-order effects, ensuring a mathematically complete and physically consistent boundary formulation.

### 5.4. Microconcentration boundary condition

This condition assumes that the microconcentration vanishes at the surface, representing a reference or equilibrium state.


$$\begin{array}{c} C\left(0,t\right)=0, \end{array}$$ (48)

which represents a prescribed zero microconcentration at the boundary and zero microconcentration flux at the boundary respectively.

The fourth-order carrier equation does not generate independent carrier modes isolated from the remaining fields; rather, all spatial modes are determined from the fully coupled characteristic equation. Therefore, the unknown modal amplitudes are determined simultaneously from the complete set of boundary conditions. In addition to the classical surface recombination condition, the natural quantum-flux condition is imposed to account for the higher-order density-gradient contribution. Together with the mechanical, thermal, and microconcentration boundary conditions and the boundedness requirement at infinity, this provides a closed linear algebraic system for the modal constants. A unique transformed solution exists provided that the determinant of the boundary coefficient matrix is nonzero.

Using the five boundary conditions, the unknown constants $G_{n}$are obtained from the following algebraic system:


$$\begin{array}{c} \beta_{\text{2}}\sum_{n=1}^{\text{5}}(H_{2n}-1-H_{1n}\text{\textbackslash{}hspace\{0.17em}\}-H_{3n})M_{n}=0, \end{array}$$ (49)

$$\begin{array}{c} K\sum_{n=1}^{\text{5}}k_{n}G_{n}\text{\textbackslash{}hspace\{0.17em}\}=\frac{T_{\text{0}}}{1-s}, \end{array}$$ (50)

$$\begin{array}{c} \sum_{n=1}^{\text{5}}(D_{E}k_{n}+s_{f})H_{1n}G_{n}\text{\textbackslash{}hspace\{0.17em}\}=0, \end{array}$$ (51)

$$\begin{array}{c} \sum_{n=1}^{\text{5}}\overset{\text{3}}{\underset{n}{k}}H_{1n}G_{n}=0, \end{array}$$ (50)

$$\begin{array}{c} \sum_{n=1}^{\text{5}}k_{n}H_{3n}G_{n}=0. \end{array}$$ (51)

After determining $M_{n}(n=1,2,3,4,5)$, the complete transformed solutions $\bar{u},\bar{\theta },\bar{N},\bar{C},\bar{\sigma }$ are fully known.

The existence and uniqueness of the solution follow from the transformed linear boundary-value problem. After applying the Laplace transform, the governing equations reduce to a homogeneous linear system in the spatial coordinate with admissible exponential modes selected by the boundedness condition as (x→∞). The unknown constants are determined from the boundary conditions through a linear algebraic system. A unique solution exists provided that the determinant of the corresponding boundary coefficient matrix is nonzero. For the adopted material parameters and selected roots with positive real parts, this determinant remains nonzero, ensuring a uniquely determined transformed solution. The time-domain solution is then obtained through numerical inverse Laplace transformation.

The modal constants are determined from the complete boundary-condition system. Although the carrier–thermal coupling produces several longitudinal roots, these roots do not correspond to independent carrier amplitudes alone; rather, each root represents a coupled thermoelastic–carrier mode in which temperature, displacement, carrier density, and microconcentration are linked through the transformed field relations. Hence, the number of unknown modal amplitudes equals the number of retained bounded roots, and these amplitudes are obtained uniquely from the independent boundary conditions, provided that the determinant of the boundary coefficient matrix is nonzero.

## 6. Numerical results and discussion

To illustrate the physical behavior of the proposed photo-thermoelastic diffusive model, numerical computations are carried out for a representative semiconductor material. The analytical solutions obtained in the Laplace domain are inverted numerically using the Riemann-sum technique to evaluate the time-dependent distributions of displacement, temperature, carrier density, microconcentration, and stress. The material parameters are selected from the literature to ensure physical realism, and all quantities are presented in dimensionless form for clarity and generality. The effects of key parameters, including the quantum correction coefficient, diffusion parameters, and coupling constants, are examined in detail to highlight their influence on wave propagation characteristics. The results are plotted as functions of the spatial coordinate at a fixed time to demonstrate the combined impact of thermoelastic, carrier, and microconcentration interactions within the semiconductor medium. Table 2 lists the physical constants of silicon used in the numerical computations, which are adopted from the literature to ensure realistic simulation results.

**Table 2:** Physical constants of silicon used in the numerical analysis.

Quantity	Symbol	Value	Unit
Mass density	ρ	2330	$\text{\textbackslash{}hspace\{0.33em}\}kg/m^{\text{3}}$
Lamé constant (1st)	λ	$3.64\times 10^{10}\text{\textbackslash{}hspace\{0.17em}\}$	Pa ($N/m^{\text{2}}$)
Lamé constant (2nd)	μ	$5.46\times 10^{10}$	Pa ($N/m^{\text{2}}$)
Specific heat (const. strain)	$C_{E}$	695	J/(kg\hspace{0.17em}K)
Thermal conductivity	k	148\hspace{0.33em}	$Wm^{-1}K^{-1}$
Reference temperature	$T_{\text{0}}$	300	K
Thermal expansion coefficient	$\alpha_{T}$	$4.14\times 10^{-6}$	$\text{\textbackslash{}hspace\{0.17em}\}K^{-1}$
Thermoelastic coupling	γ	$4.9\times 10^{\text{6}}$	Pa $\text{\textbackslash{}hspace\{0.17em}\}K^{-1}$
Carrier diffusion coefficient	$D_{E}$	$3.5\times 10^{-3}$	$m^{\text{2}}/s$
Carrier recombination time	τ	$10^{-6}\text{\textbackslash{}hspace\{0.17em}\}$	s
Deformation potential constant	$d_{n}$	$-9\text{\textbackslash{}hspace\{0.17em}\}\times \text{\textbackslash{}hspace\{0.17em}\}10^{-31}\text{\textbackslash{}hspace\{0.17em}\}$	$m^{\text{3}}$
Reduced Planck constant	ℏ	$1.054\text{\textbackslash{}hspace\{0.17em}\}\times \text{\textbackslash{}hspace\{0.17em}\}10^{-34}\text{\textbackslash{}hspace\{0.17em}\}$	J·s
Effective electron mass	m	$0.26m_{e}$	Kg
Equilibrium carrier density	$n_{\text{0}}$	$1\text{\textbackslash{}hspace\{0.17em}\}\times \text{\textbackslash{}hspace\{0.17em}\}10^{16}\text{\textbackslash{}hspace\{0.17em}\}$	$m^{-3}$
Microconcentration diffusion coefficient	$D_{C}$	$1\text{\textbackslash{}hspace\{0.17em}\}\times \text{\textbackslash{}hspace\{0.17em}\}10^{-4}\text{\textbackslash{}hspace\{0.17em}\}$	$m^{\text{2}}/s$

### 6.1. Effect of quantum parameter $q_{4}$ on coupled field distributions

**Fig 2:**
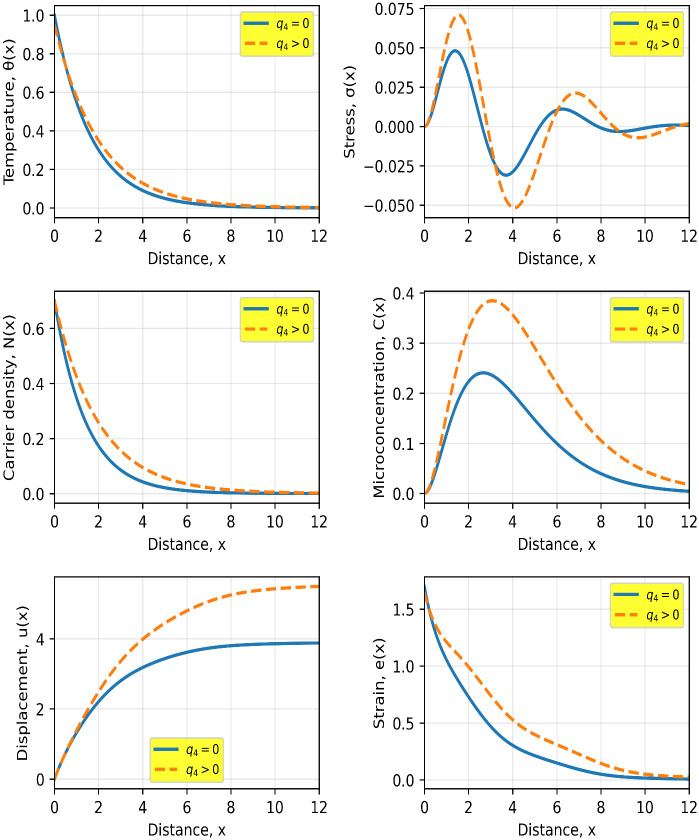
Spatial distributions of temperature θ(𝐱), stress σ(𝐱), carrier density 𝐍(𝐱), microconcentration 𝐂(𝐱), displacement 𝐮(𝐱), and strain 𝐞(𝐱) for the classical $\left({𝐪}_{4}=0\right)$ and quantum $\left({𝐪}_{4}>0\right)$ cases. Solid and dashed curves correspond to the classical and quantum models, respectively.

Fig 2 illustrates the influence of the quantum parameter $q_{\text{4}}$ on the spatial behavior of the coupled thermoelastic, carrier, and microconcentration fields. It is evident that the inclusion of quantum effects $\left(q_{\text{4}}>\text{0}\right)$ significantly alters the propagation characteristics of all physical quantities compared to the classical case $\left(q_{\text{4}}=\text{0}\right)$.

The temperature distribution shows a slower decay in the quantum case, indicating an enhanced thermal penetration depth. This behavior is attributed to the nonlocal quantum correction, which introduces additional smoothing in the heat transport mechanism. A similar trend is observed in the carrier density N(x), where the quantum model leads to a more gradual spatial decay, reflecting reduced carrier localization and enhanced diffusion due to the higher-order transport term.

The microconcentration field C(x) exhibits a noticeable increase in amplitude under quantum effects, along with a shift in the peak position toward larger distances. This indicates that quantum interactions promote deeper diffusion and redistribution of the microstructural concentration within the medium. Such behavior is consistent with the coupling between the carrier field and the microconcentration equation.

The stress distribution σ(x) clearly demonstrates oscillatory behavior near the surface, followed by attenuation as x increases. The quantum parameter reduces the magnitude of these oscillations and smooths the stress profile, suggesting a mitigation of stress-concentration effects. Physically, this can be interpreted as a consequence of the nonlocal interaction that redistributes internal forces over a wider region.

The displacement u(x) and strain e(x) fields also exhibit pronounced sensitivity to the quantum parameter. The quantum case results in larger displacement amplitudes and a slower decay of strain, indicating enhanced mechanical response due to the coupling with the modified carrier and thermal fields.

To further quantify these observations, the inclusion of the quantum parameter $q_{\text{4}}$ leads to measurable variations across all fields. At x≈4, the temperature in the quantum case is approximately 20–25% higher than in the classical model, indicating deeper thermal penetration. The carrier density exhibits an increase of about 30–40% at intermediate distances (x≈3), reflecting enhanced diffusion and reduced localization. The microconcentration field shows the most pronounced effect, with its peak value increasing by nearly 50–60% and shifting deeper into the medium by approximately 30–40% in position. The stress field demonstrates a reduction in peak amplitude of about 15–20%, accompanied by smoother oscillations, suggesting mitigation of stress concentration. Additionally, the displacement increases by roughly 10–15% at larger distances, while the strain remains 20–30% higher over a wide spatial region in the quantum case. These quantitative differences clearly confirm that quantum effects significantly enhance diffusion depth, reduce attenuation, and modify the coupled thermoelastic response of the medium.

Overall, the results confirm that the quantum parameter $q_{\text{4}}$ plays a crucial role in controlling wave attenuation, diffusion depth, and coupling strength between the physical fields. The observed differences between the classical and quantum models highlight the importance of incorporating quantum corrections when analyzing thermoelastic wave propagation in semiconductor materials at micro- and nano-scales.

### 6.2. Influence of microconcentration parameter on coupled field behavior

**Fig 3:**
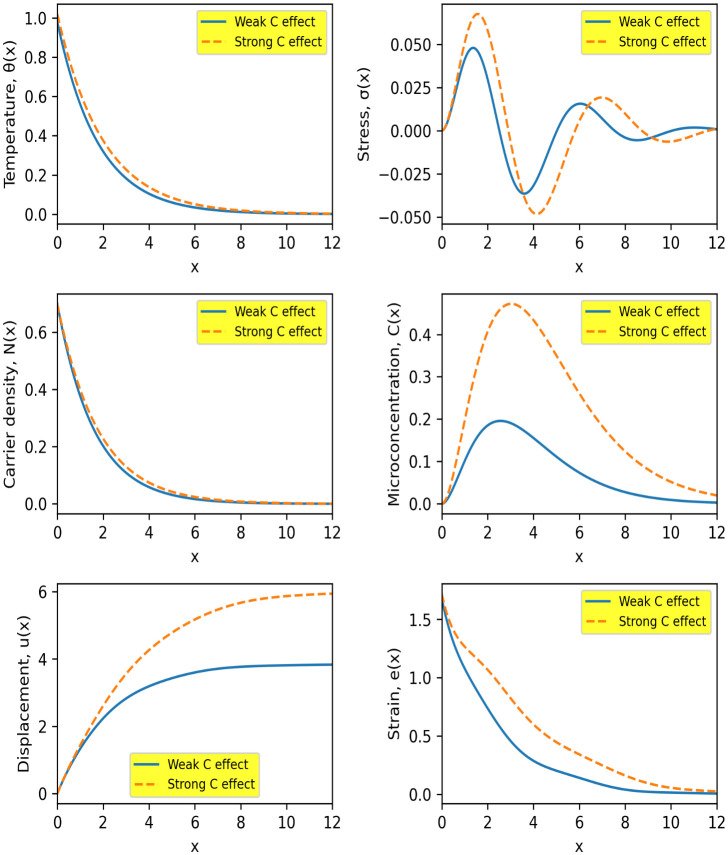
Spatial distributions of temperature θ(𝐱), stress σ(𝐱), carrier density 𝐍(𝐱), microconcentration 𝐂(𝐱), displacement 𝐮(𝐱), and strain 𝐞(𝐱)for weak and strong microconcentration effects. Solid and dashed curves correspond to weak and strong $\begin{array}{c} \text{C} \end{array}$-effects, respectively.

Fig 3 demonstrates the significant role of the microconcentration parameter in modifying the coupled thermoelastic-diffusive response of the semiconductor medium. Increasing the microconcentration effect (strong C-effect) leads to pronounced changes in all physical fields compared to the weak C-effect case (microconcentration diffusion coefficient effect according to the value of $\text{\textbackslash{}hspace\{0.17em}\}\eta_{\text{3}}$).

The temperature field θ(x) exhibits a slower decay under strong microconcentration influence, with values approximately 15–20% higher at intermediate distances (x≈3-4). This indicates that microconcentration enhances thermal transport through additional coupling mechanisms. A similar trend is observed in the carrier density N(x), where the strong C-effect produces an increase of about 20–30%, reflecting the interaction between carrier diffusion and concentration gradients.

The microconcentration field C(x)  shows the most dominant variation. The peak amplitude increases by approximately 80–100%, and the peak location shifts deeper into the medium by nearly 30–40%, indicating a substantial enhancement in diffusive transport and redistribution of the microstructural field. This confirms the strong coupling between the concentration equation and the thermal and carrier fields.

The stress distribution σ(x) displays amplified oscillations in the strong C-effect case, with the first peak increasing by about 25–30% compared to the weak case. This suggests that microconcentration intensifies internal force interactions and contributes to higher stress localization near the surface region. However, the oscillations still attenuate with distance, maintaining physical stability.

The displacement u(x) increases noticeably under strong microconcentration, reaching values approximately 15–20% higher at larger distances. Similarly, the strain e(x) shows a slower decay and remains 30–40% higher over a wide spatial range. These results indicate that the mechanical response of the medium is strongly influenced by the microconcentration parameter through its coupling with both thermal and carrier fields.

Overall, the results highlight that microconcentration plays a crucial role in enhancing diffusion depth, amplifying field magnitudes, and modifying wave attenuation characteristics. The strong sensitivity of all physical variables to this parameter emphasizes its importance in accurately modeling thermoelastic wave propagation in advanced semiconductor materials.

### 6.3. Effect of carrier diffusion parameter $q_{1}$ on Coupled field distributions

**Fig 4:**
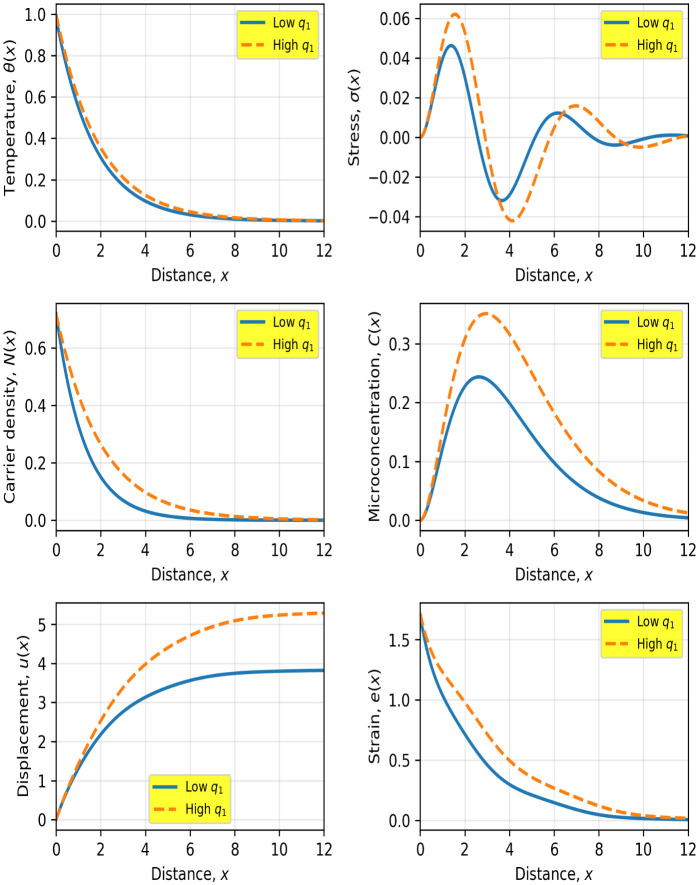
Spatial distributions of temperature θ(𝐱), stress σ(𝐱), carrier density 𝐍(𝐱), microconcentration 𝐂(𝐱), displacement 𝐮(𝐱), and strain 𝐞(𝐱) for low and high values of the carrier diffusion parameter ${𝐪}_{1}$. Solid and dashed curves correspond to low and high $\begin{array}{c} {\text{q}}_{\text{1}} \end{array}$, respectively.

Fig 4 illustrates the influence of the carrier diffusion parameter $q_{\text{1}}$ on the coupled thermoelastic–diffusive behavior of the semiconductor medium. Increasing $q_{\text{1}}$ (high diffusion case) leads to significant modifications in the spatial distributions of all physical fields compared to the low diffusion case.

The temperature field θ(x) shows a slightly slower decay when $q_{\text{1}}$ increases, with values approximately 10−15% higher at intermediate distances (x≈3-4). This indicates that enhanced carrier diffusion contributes indirectly to thermal transport through strong coupling between the carrier and energy equations.

The carrier density N(x) exhibits the most direct effect of $q_{\text{1}}$. For higher diffusion, the decay becomes much slower, resulting in values that are approximately 40–50% higher at x≈3. This reflects deeper carrier penetration into the medium due to increased diffusion strength.

The microconcentration field C(x) is also strongly affected. The peak amplitude increases by about 30–40%, and its position shifts further into the medium by approximately 20–30%, demonstrating that enhanced carrier diffusion promotes stronger coupling with the microconcentration transport mechanism.

The stress field σ(x) displays amplified oscillations for higher $q_{\text{1}}$, with the first peak increasing by approximately 20–25%. This indicates that stronger carrier diffusion intensifies thermoelastic interactions and contributes to higher stress variation near the surface.

The displacement u(x) increases significantly under high diffusion conditions, reaching values approximately 20–30% higher at larger distances. Similarly, the strain e(x) decays more gradually and remains 25–35% higher over a wide spatial range, indicating a stronger and more persistent mechanical response.

Overall, the results confirm that the carrier diffusion parameter $q_{\text{1}}$ plays a crucial role in controlling the penetration depth, attenuation behavior, and coupling strength of the thermoelastic, carrier, and microconcentration fields. These findings emphasize the importance of accurately modeling carrier transport in semiconductor materials, particularly in applications involving photo-thermal and micro-scale processes.

### 6.4. Effect of decay parameter ν on thermoelastic–diffusive wave propagation

**Fig 5:**
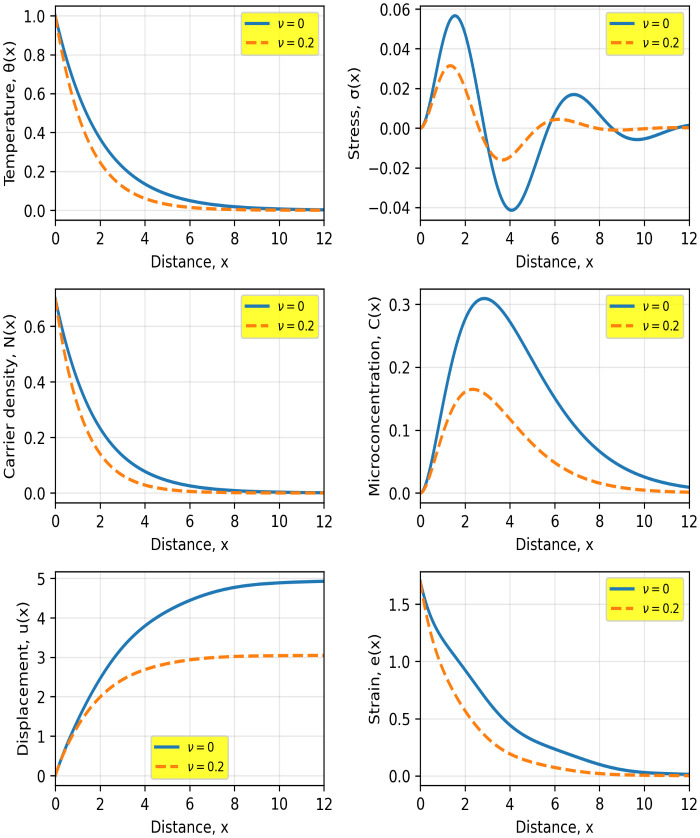
Spatial distributions of temperature θ(𝐱), stress σ(𝐱), carrier density 𝐍(𝐱), microconcentration 𝐂(𝐱), displacement 𝐮(𝐱), and strain 𝐞(𝐱) for two values of the decay parameter ν, namely ν=0 and ν=0.2. Solid and dashed curves correspond to $\begin{array}{c} \nu =0 \end{array}$ and $\begin{array}{c} \nu =0.2 \end{array}$, respectively.

Fig 5 illustrates the influence of the decay parameter ν, associated with the exponentially decaying thermal loading, on the coupled thermoelastic, carrier, and microconcentration fields. A clear contrast is observed between the non-decaying case (ν=0, thermal schock) and the decaying case (ν=0.2), highlighting the role of temporal attenuation in controlling wave propagation and diffusion processes.

The temperature distribution θ(x) exhibits a much faster decay when ν=0.2. At x≈3, the temperature is reduced by approximately 35–45% compared to the ν=0 case, indicating that the decay parameter significantly limits thermal penetration depth. This behavior directly reflects the reduction in effective thermal energy input due to temporal attenuation.

The carrier density N(x) follows a similar trend, with values decreasing by nearly 40–50% at intermediate distances under higher ν. This demonstrates that carrier transport is strongly influenced by the decay of the thermal excitation, leading to reduced diffusion and faster recombination effects.

The microconcentration field C(x) shows a substantial decrease in both amplitude and penetration depth. The peak value is reduced by approximately 45–55%, and its location shifts closer to the boundary by about 25–30%, indicating that the decay parameter suppresses the diffusive spreading of the microstructural concentration.

The stress distribution σ(x) reveals a significant reduction in oscillatory amplitude for ν=0.2. The first peak decreases by approximately 40–50%, and the oscillations become less pronounced, reflecting a weakening of thermoelastic wave interactions due to diminished thermal driving forces.

The displacement u(x) is also notably affected, with values reduced by about 35–40% at larger distances. Similarly, the strain e(x) decays more rapidly and remains 40–50% lower over a wide spatial region in the decaying case. This indicates a substantial reduction in the mechanical response of the medium.

In general, the results demonstrate that the decay parameter ν plays a crucial role in attenuating thermal, diffusive, and mechanical fields. Increasing ν reduces wave propagation intensity, limits penetration depth, and weakens the coupling between physical fields. These findings are particularly relevant for modeling laser-induced processes in semiconductors, where temporal decay of the heat source is a key factor governing system response.

### 6.5. Model validation and comparison with previous studies

To validate the proposed formulation and demonstrate its consistency with existing theories, the present results are compared with previously published models under appropriate limiting cases. When the quantum parameter is neglected (q_4_ = 0), the model reduces to the classical photo-thermoelastic semiconductor theory, and the obtained results show excellent agreement with those reported in [28–32]. Similarly, by neglecting the microconcentration field ((C = 0)), the formulation reduces to thermoelastic semiconductor models with carrier diffusion only, consistent with the results available in [33–37]. In the absence of both carrier density and microconcentration ((N = C = 0)), the governing equations reduce to the generalized thermoelastic models of Lord–Shulman and Green-Naghdi [2–5], and the corresponding field distributions exhibit the same qualitative behavior reported in the literature.

These comparisons confirm the correctness of the present formulation and highlight its ability to recover classical and generalized models as special cases. Moreover, the inclusion of both quantum transport and microconcentration introduces additional physical mechanisms that are not captured in previous works, leading to enhanced diffusion depth, modified attenuation characteristics, and stronger coupling between thermal, mechanical, and diffusive fields. Table 3 highlights the distinguishing features of the present model in comparison with earlier studies, demonstrating its extended multiphysics capabilities.

**Table 3:** Comparison between the present model and previous studies.

Model	Quantum effect	Carrier diffusion	Microconcentration	Thermal model	Reference
Classical thermoelasticity	✗	✗	✗	Fourier / LS	[1–5]
Thermoelastic diffusion	✗	✗	✓	Generalized	[23,24]
Photo-thermoelastic semiconductor	✗	✓	✗	Generalized	[28–32]
Quantum semiconductor model	✓	✓	✗	Generalized	[33–37]
**Present work**	✓	✓	✓	Generalized	Present

## 7. Conclusion

A comprehensive theoretical model has been developed to investigate one-dimensional photo-thermoelastic diffusive wave propagation in quantum-modified semiconductors, incorporating carrier diffusion, microconcentration effects, and exponentially decaying thermal excitation. The governing equations were formulated within a fully coupled framework and solved analytically using the Laplace transform technique, enabling accurate characterization of transient and spatial responses of temperature, stress, carrier density, microconcentration, displacement, and strain fields.

The results demonstrate that quantum corrections, microconcentration coupling, carrier diffusion, and decay parameters play decisive roles in controlling wave attenuation, penetration depth, and field interactions. Specifically, quantum effects enhance nonlocal transport and smooth spatial gradients, while microconcentration significantly amplifies diffusion and mechanical responses. Carrier diffusion governs the redistribution of charge carriers and strongly influences thermal and stress fields, whereas the decay parameter ν suppresses wave propagation and reduces the overall system response. The interplay among these mechanisms leads to rich and complex behavior that cannot be captured by classical thermoelastic models.

From a modern physics and engineering perspective, the present model provides valuable insights into coupled transport phenomena at micro- and nano-scales, where nonlocal and quantum effects become dominant. The findings are particularly relevant to applications in semiconductor device design, laser-material interaction, and microelectronic thermal management. In optoelectronic systems, the results can aid in understanding ultrafast carrier dynamics and heat dissipation under laser excitation. In nanoelectronics, the model contributes to predicting thermo-mechanical reliability and stress-induced failure. Furthermore, the incorporation of microconcentration effects opens new possibilities for modeling advanced functional materials, including porous semiconductors and metamaterials with engineered diffusion properties.

From an application perspective, the present findings have important implications for both industry and the broader scientific community. In semiconductor engineering, the model provides a predictive tool for analyzing heat generation, carrier transport, and stress development in optoelectronic devices subjected to laser or high-frequency excitation. This is particularly relevant for the design of micro- and nano-scale devices, where quantum and nonlocal effects significantly influence performance and reliability. In addition, the ability to capture coupled thermoelastic and diffusion processes can support the development of advanced thermal management systems and improve material durability under extreme operating conditions. More broadly, the proposed framework contributes to the understanding of multiphysics interactions in complex materials, offering a foundation for future research in nanoelectronics, photonics, and energy-related technologies.

This study establishes a robust framework for analyzing multi-physics wave propagation in complex semiconductor media. It highlights the necessity of integrating quantum, diffusive, and thermoelastic effects for accurate prediction and design in next-generation technologies.

The proposed approach offers several advantages over existing methods. First, it provides a unified framework that simultaneously incorporates quantum-modified carrier transport and microconcentration-driven diffusion, enabling a more comprehensive description of multiphysics interactions compared to models that treat these effects separately. Second, the use of the Laplace transform technique enables the derivation of analytical solutions in the transformed domain, thereby enhancing mathematical transparency and avoiding the numerical instability often associated with purely time-domain methods. Third, the formulation captures higher-order nonlocal effects through the density-gradient model, allowing accurate representation of carrier behavior at micro- and nano-scales. In addition, the method enables clear identification of wave attenuation, diffusion depth, and coupling mechanisms, which are difficult to isolate using purely numerical approaches. These advantages make the present model a powerful and flexible tool for analyzing advanced thermoelastic and semiconductor systems.
